# Interprofessional simulation training in acute pediatrics using rapid cycle deliberate practice (ip-star)

**DOI:** 10.1186/s12909-025-08302-4

**Published:** 2025-12-29

**Authors:** Manish Kumar, Mahima Mittal, Seema Shankarsingh Chavan, Vimal Krishnan S, John H.V. Gilbert, Elsa Sanatombi Devi

**Affiliations:** 1 MAHE- FAIMER International Institute for Leadership in Interprofessional Education (M-FIILIPE), Manipal, Karantaka India; 2 AIIMS Gorakhpur, Gorakhpur, Uttar Pradesh India; 3 College of Nursing, AIIMS Gorakhpur, Gorakhpur, India; 4 Kasturba Medical College, Manipal, Manipal Academy of Higher Education (MAHE), Manipal & Centre for Resuscitation, Acute Care and Simulation Training (CReST), MAHE, Manipal, Karnataka India; 5 University of British Columbia & Dr. T.M. Pai Chair in Interprofessional Education and Care, Manipal Academy of Higher Education (MAHE), Manipal, India; 6 Medical Surgical Nursing Department, Manipal College of Nursing, Manipal Academy of Higher Education, Manipal & Director MAHE FAIMER International Institute for Leadership in Interprofessional Education, Manipal Academy of Higher Education (MAHE), Manipal, Karnataka India

**Keywords:** Interprofessional education, Simulation training, TeamSTEPPS, Rapid cycle deliberate practice, Pediatrics, Undergraduate education, Quality education

## Abstract

**Background:**

Undergraduate curriculums of healthcare professionals, designed as discipline-specific silos, focus more on knowledge and less on skills, and minimally on nontechnical skills and team dynamics. Lack of prior team training often results in poor team dynamics in face of the challenges of managing high stakes crisis scenarios of acute pediatrics. This study aimed to evaluate the effectiveness of Interprofessional Simulation Education (IPSE) using Rapid Cycle Deliberate Practice (RCDP) and the Team Strategies and Tools to Enhance Performance and Patient Safety (TeamSTEPPS) framework for undergraduate medical and nursing students.

**Methods:**

A one-group pre-post study design was adopted with intervention consisting of in-situ interprofessional simulation of a pediatric cardiac arrest scenario using RCDP methodology, structured within the TeamSTEPPS framework. Assessment measures included multiple choice question (MCQ) based test, team Objectively Structured Clinical Examination (OSCE), Team Performance Observation Tool (TPOT), and self-perceived confidence surveys. Participants’ feedback were collected regarding the interprofessional training experience.

**Results:**

A total of 72 third-year students, comprising 46 MBBS and 26 BSc Nursing students, participated in this study conducted across 12 interprofessional simulation training sessions. The intervention led to significant improvements across all measured domains. Knowledge acquisition, assessed using MCQs, showed a marked increase, with post-test scores demonstrating significantly higher median (IQR) values compared to pre-test scores pre-test median 6 (2) vs. post-test median 8 (2), *p* < 0.01). Similarly, team-based clinical skills, evaluated through Team OSCE scores, improved substantially following the training pre-training median 6 (2.25) vs. post-training median 17 (2), *p* < 0.002).

Assessment done using TPOT also demonstrated statistically significant enhancement across all 5 domains, i.e. team structure, communication, leadership, situational monitoring and mutual support. Furthermore, learners reported a notable increase in self-perceived confidence related to managing pediatric emergencies. Feedback on sessions’ content, interprofessional collaboration development, skill acquisition and educational value showed high learner endorsement.

**Conclusions:**

The novel combination of RCDP methodology within the TeamSTEPPS framework in IPSE can help in development of effective individual skills and teamwork competencies among novice learners. Future studies should examine long-term retention of these skills and their transfer to clinical practice.

**Supplementary Information:**

The online version contains supplementary material available at 10.1186/s12909-025-08302-4.

## Background

Undergraduate curriculums of healthcare professionals, especially in Low- and Middle-Income Countries (LMIC), consist of didactics and apprenticeship models which focus more on knowledge and less on technical skills [1]. The emphasis is even lesser on non-technical skills such as leadership, situational awareness, communication and team dynamics [2]. Further, these educational programs are siloed where learners have minimal interactions with peers from other healthcare professions, even though eventually, they are expected to work as an interprofessional team after their program completion [3]. This discordance between training methodology and practice expectations represents a significant challenge in health professions education.

Interprofessional education (IPE), where students from different health professions “learn with, from, and about each other to improve collaboration and the quality of care and services” [4] is a solution for this challenge. IPE, despite being recognized by the World Health Organization (WHO) and numerous other accreditation agencies, as an essential component of health professions education, has sparsely been leveraged for team training in undergraduate curriculums, more so, for pediatric critical care training. This lack of interprofessional team training in acute pediatric care settings, where healthcare professionals must work seamlessly as a team, applying both technical and non-technical skills in high stakes crisis scenarios, often translates into poor team performance and sub optimal health outcomes.

Simulation Based education (SBE) has emerged as an indispensable educational modality in healthcare [5]. Multiple recent studies in nursing education have demonstrated that SBE enhances learners’ self-confidence, satisfaction, and overall clinical performance [6–9]. Similarly, in interprofessional settings, simulation training has been shown to improve crisis resource management skills and team performance [10]. Debriefing, which is the proverbial core of SBE, traditionally and especially in interprofessional simulation education (IPSE), takes place at the end of simulation for guided reflections by learners. Rapid Cycle Deliberate Practice (RCDP), first introduced by Elizabeth Hunt, is a novel debriefing methodology in simulation training that allows the learners to repetitiously perform a simulation scenario, segmented into progressively complex stages, with frequent and succinct micro-debriefs by faculty [11]. This allows the learners to incorporate feedback focusing on specific areas of weakness for continuous improvement. Once acceptable performance is obtained, the simulation shifts into next, more complex stage to continue an iterative process of mastery learning through deliberate practice. There is considerable evidence regarding utility of RCDP in teaching procedural skills and algorithmic skill protocols in single profession groups [11, 12]. However, the evidence regarding use of RCDP for augmenting non-technical skills such as team performance is relatively sparse [13]. These non-technical competencies, though critical, are often under-addressed in simulation debriefings as facilitators often struggle to objectively evaluate team dynamics. Team Strategies and Tools to Enhance Performance and Patient Safety (TeamSTEPPS) is an evidence-based framework developed by the Agency for Healthcare Research and Quality (AHRQ) for optimal team performance and enhanced patient safety [14]. The four core components of this framework – communication, leadership, situational monitoring and mutual support are presented as teachable – learnable skills. Hence, an emerging body of evidence from SBE has integrated the structured TeamSTEPPS framework to allow for an objective evaluation of team dynamics and enable facilitators to deliver focused, actionable feedback [15]. Despite a growing body of evidence supporting the feedback-intensive RCDP and robust TeamSTEPPS framework as effective pedagogical tools individually, their combined use for undergraduate interprofessional healthcare education remains largely unexplored. Integration of these two approaches in IPSE presents an innovative model with significant latent potential for teaching interprofessional learners technical and non-technical skills related to acute pediatric care.

In this context, the present study aimed to evaluate the effectiveness of a simulation-based interprofessional training intervention integrating Rapid Cycle Deliberate Practice and the TeamSTEPPS framework, in enhancing undergraduate medical and nursing students’ knowledge, teamwork, communication, and confidence in managing acute pediatric cardiac arrest scenarios.

## Methods

### Study setting and design

The study was conducted at a tertiary care healthcare institute in Northern India. The institute has no formal IPE curriculum, and the present study was designed as a pilot educational innovation project in IPE. The interprofessional team for this project included faculty from the department of Pediatrics and College of Nursing. During informal interactions, conducted as part of needs assessment, both medical and nursing student groups cited limited exposure to interprofessional team training. The IPE team for this project in their deliberations also appreciated the need for a standardized curriculum for team training in acute pediatric care.

The study employed quasi-experimental, one group pre-post interventional design with the objective of assessing the effectiveness of interprofessional simulation-based team training on knowledge, skills, and team performance of third year Bachelor of Medicine, Bachelor of Surgery (MBBS) and Bachelor of Science (BSc) Nursing students in simulated pediatric cardiopulmonary resuscitation (CPR) scenario. No formal sample size calculation was performed. The study included all eligible participants available during the intervention period, following a convenience sampling approach based on cohort availability.

### Intervention

Educational intervention consisted of a two-hour workshop structured to provide systematic progression from foundational knowledge assessment to skill enhancement and team-based simulation training. The IP-STAR module was designed as an in-situ simulation within the clinical environment to promote realistic, team-based learning in acute pediatric care. A low-technology PRESTAN^®^ Infant Manikin was coupled with enhanced environmental fidelity using hospital props such as standard pediatric ward equipment, hospital clothing, and crash cart trolleys. Additionally, to ensure simulation safety, this IPSE module adopted practices such as use of empty medication vials filled with saline and clearly labeled “for simulation purposes only” for simulating drug administration procedures and return of equipment used in simulation for clinical use after appropriate decontamination [16].

**Fig. 1:**
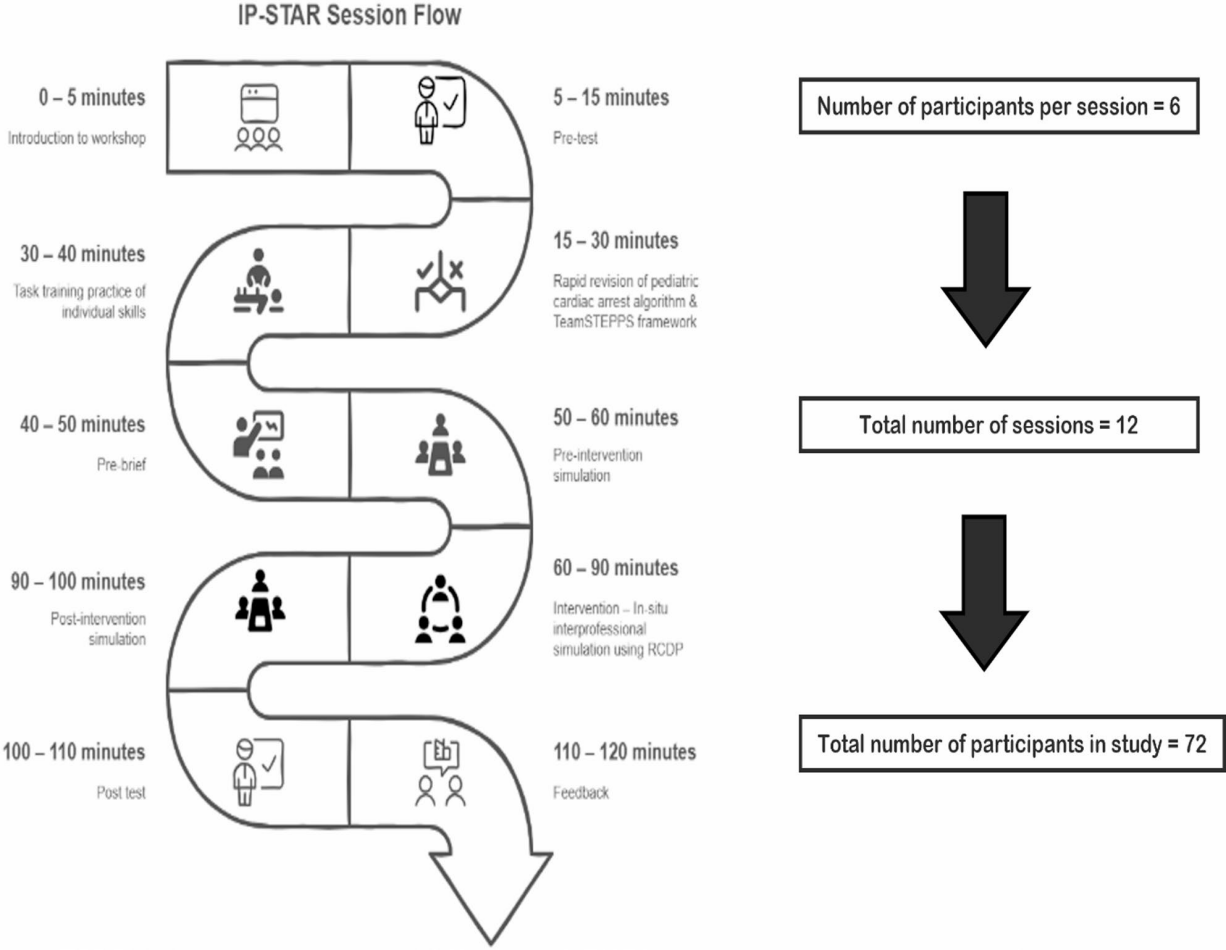
IP-STAR Session Flow

Each session included 3–4 third year MBBS and 2–3 BSc Nursing students. The participants were novice learners with basic clinical algorithm and skills training in their respective programs but limited exposure to interprofessional teamwork and acute care scenarios. Introduction and pretest were followed by a quick recap of pediatric cardiac arrest algorithm and TeamSTEPPS framework. Learners were, then, allowed to practice or task train individual skills like airway management, bag and mask ventilation and chest compressions. Thereafter, participants were prebriefed to lay out basic assumptions, ensure confidentiality and psychological safety, and to establish fiction contracts [17]. Participants were oriented to in-situ simulation setting, manikin, equipment and clinical context of simulation scenario. During the prebrief, the learners were asked to identify roles of various team members in resuscitation scenario. It was also clarified that role assignments should be done through a quick discussion between the team members and the roles of nursing students should not be restricted to medications and documentation only. Prebrief was followed by pre-intervention simulation in which facilitators completed pre intervention team Objectively Structured Clinical Examination (OSCE) scores, TeamSTEPPS Team Performance Observation Tool (TPOT) scoring. Then, core intervention in the form of interprofessional simulation training using RCDP was carried out enabling iterative improvement through immediate feedback. A post-intervention simulation was conducted using the same assessment tools to measure team performance gains. This was followed by a post-test and feedback to assess improvement in knowledge and gather participants’ reflections. The flow of IP-STAR session is summarized in Fig. 1.

### Simulation scenario and RCDP rounds

The simulation scenario was titled “Pediatric Cardiac Arrest: Pulseless Electrical Activity with Hypovolemic Shock” and described a case of 8-month-old infant presenting in an unresponsive state, pulseless and in apnea, following a history of diarrhea, vomiting, and poor feeding (Additional File 1). The in-situ simulation scenario was designed to reinforce both technical and non-technical skills. The scenario had embedded participants to enhance fidelity and to address communication competencies. The simulation content was customized to remain relevant to competencies described in both MBBS and BSc Nursing curriculums. Additionally, six domains described in Canadian Interprofessional Health Collaborative (CIHC) Competency Framework for Advancing Collaboration – relationship focused care, team communication, role clarification, team functioning, team differences processing and collaborative leadership [18], were referred to while designing the simulation scenario.

**Fig. 2:**
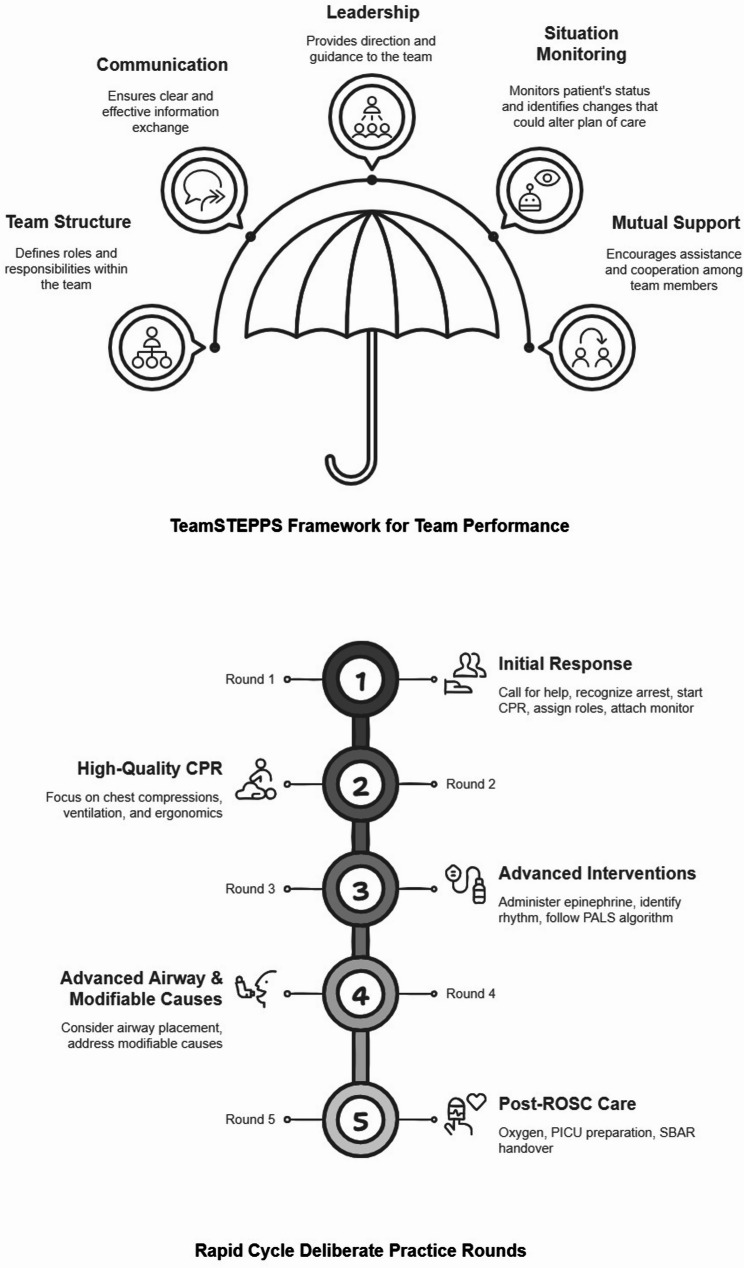
TeamSTEPPS Framework for Team Performance and RCDP Rounds

The simulation scenario was segmented into five structured RCDP rounds, each focused on predetermined learning objectives. Round 1 addressed the recognition of cardiac arrest, calling for help, immediate initiation of CPR, role allocation, and monitor attachment. Round 2 emphasized high-quality CPR including optimal ergonomics and effective bag-mask ventilation. In Round 3, learners were expected to administer epinephrine promptly, perform rhythm checks, and follow the Pediatric Advanced Life Support (PALS) algorithm. Round 4 focused on airway management and identifying potentially reversible causes of cardiac arrest, while Round 5 involved post-resuscitation care and Situation-Background-Assessment-Recommendation (SBAR) handover. In addition to technical skills, a demonstration of optimal team performance based on elements of TeamSTEPPS framework i.e. team structure, communication, leadership, situational monitoring and mutual support was the overarching objective. This amalgamation of technical and nontechnical skills during different RCDP rounds is summarized in Fig. 2.

As per RCDP methodology, once learners demonstrated reasonable proficiency in a given round, the scenario progressed to the next round introducing additional clinical complexity and need for further interventions. However, each iteration commenced from Round 1, thereby reinforcing prior learning and allowing students to build competence through repeated practice. RCDP debrief guidance during simulation scenario was pre-decided in form of ‘Hard stops’ and ‘Soft Stops’. Hard stops were for actions which were directly linked to learning objectives of each round and must be performed in the recommended way, while soft stops were for actions where variability of performance is allowed [12]. While soft stops mostly related to team dynamics, communication and sharing of mental models, certain critical elements of team performance such as assembling a team, role allocation, clear messages, monitoring of patient status and mutual respect constituted hard stops in addition to primary learning objectives for each round. The objectives, presentation and relevance of simulation scenario script for transfer of knowledge and skills in interprofessional team training were reviewed and validated with scale content validity index (s-CVI) of 0.93, by five external experts (three nursing and two medical faculty) with more than five years of experience as simulation instructors.

### Data collection

Data were collected at two time points – pre intervention and post intervention. The pretest and posttest assessments were conducted on the same day, immediately before and after the simulation-based intervention, respectively. Assessment instruments included test with MCQ scores for knowledge, team OSCE scores for skills, TPOT scores for team performance, and learner survey for self-perceived confidence in delivery of acute pediatric care in interprofessional settings. Participant feedback was also collected regarding the interprofessional training experience at the end of the module.

### Instruments

#### Knowledge

A questionnaire with 10 MCQs was developed to assess the participants’ knowledge (Additional File 2). This assessment tool covered both clinical content related to pediatric cardiac arrest management and teamwork concepts. Content validity was evaluated across three domains: relevance, clarity, and appropriateness with the help of 6 external experts (2 intensivists, 1 pediatrician, 2 nursing experts). The s-CVI for relevance, clarity, and appropriateness were 0.96, 0.93, and 0.96, respectively, indicating high overall content validity.

#### Skills and team performance

For assessment of skills, a Team OSCE checklist was developed. This team OSCE had 20 items evaluating critical skills as individual team members of pediatric resuscitation team along with elements of good team dynamics (Additional File 2). Each item was scored on a binary 1-point scale: 1 for correct and complete demonstration of the skill, and 0 for incorrect or absent performance. The 6 external experts who validated pre/posttest also validated the team OSCE. This instrument had high content validity with the s-CVI for relevance, clarity, and appropriateness being 1, 0.97, and 0.97, respectively.

Concomitantly, teams were assessed on their performance using the TeamSTEPPS framework based TPOT developed by AHRQ. TPOT evaluates teams across five key domains: team structure, leadership, situational monitoring, mutual support, and communication. This assessment tool uses a 5-point Likert scale ranging from very poor to excellent, allowing observers to rate how well teams demonstrate essential competencies across the key teamwork domains during real or simulated clinical events.

The binary OSCE checklist evaluated individual and team performance against a predefined criteria while behaviorally anchored TPOT complemented it by providing an objective assessment of team behaviors during simulation. This dual approach enabled raters to identify completion of essential tasks and presence of effective team dynamics.

Team performance assessment using Team OSCE and TPOT was done by two raters in all sessions during this project. In order to ensure objectivity and consistency, the raters were trained using mock simulations during the pilot phase of the project prior to data collection. The training involved joint review and scoring of pilot simulation videos followed by consensus discussions to standardize interpretation of checklist items and performance anchors. Scoring rubrics of Team OSCE were clearly defined and the use of TPOT, which is a behaviorally anchored rating scale also helped in reducing subjectivity.

#### Self-perceived confidence

Participants were asked to complete a survey regarding self-perceived confidence before and after the intervention. This 10-item survey assessed learners’ self-perceived confidence in executing critical skills related to pediatric CPR and fulfilling essential interprofessional collaborative practice competencies (Additional File 2). The survey was adapted from similar instruments used in previous studies of interprofessional simulation education [19].

#### Participant feedback

Postworkshop feedback from participants was collected in which the participants were asked to indicate their level of agreement with statements on a Likert scale ranging from strongly disagree to strongly agree (Additional File 2). The statements were on session content, interprofessional collaboration, skill development and educational value of IP-STAR.

### Data analysis

Dichotomous or categorical data are presented as absolute numbers and percentages. The Shapiro-Wilk test was used to assess the distribution of continuous variables. Continuous variables with normal distribution are presented as mean with standard distribution while non-normal distribution is presented as median and interquartile range (IQR). Based on distribution, paired T test or Wilcoxon signed-rank test was used to compare pre- and post-intervention scores while McNemar’s Test was used to compare paired nominal data. All statistical analyses were conducted using Jamovi (version 2.6.26), and a p-value of less than 0.05 was considered statistically significant.

### Ethical considerations

The study was approved by the Institutional Human Ethics Committee. Written informed consent was obtained from all participants after informing them about the study procedure. Participation in IP-STAR training was voluntary, and students were also informed that their participation or non-participation will have no bearing on their academic assessment under the Institute’s curriculum. The study adhered to the Declaration of Helsinki. All arrangements were ensured that the in-situ simulation training did not affect routine patient care and best practices related to simulation safety were followed [16].

## Results

A total of 72 third-year students, comprising 46 MBBS and 26 BSc Nursing students, participated in this study conducted across 12 interprofessional simulation training sessions. The median age of the participants was 22 years, with 37 female and 35 male students.

### Knowledge

Knowledge acquisition showed a marked increase, with post-test scores demonstrating significantly higher median (IQR) values compared to pre-test scores - pre-test median 6 (2) vs. post-test median 8 (2), *p* < 0.01. The proportion of participants answering correctly increased significantly across most key domains following the intervention. Cognitive gains in some key pediatric cardiac arrest domains are summarized in Table 1.

**Table 1:** Proportion of participants answering correctly across key knowledge domains (Pre- vs. Post-Intervention,)

Knowledge domain	Pre-Intervention Correct, *n* (%)	Post-Intervention Correct, *n* (%)	*p*-value^*^
Identification of cardiac arrest	71 (98.6)	71 (98.6)	1.00
Depth of chest compressions	57 (79.2)	70 (97.2)	< 0.001
Dose of epinephrine	57 (79.2)	68 (94.4)	0.012
Identification of cardiac arrest rhythm	38 (52.8)	66 (91.7)	< 0.001
Defibrillation Dose	38 (52.8)	53 (73.6)	0.002

*n* = 72

*McNemar Test

#### Skills and team performance

Team OSCE checklist had 20 items, scored on binary 1 point scale per item, assessing critical skills as individual team members of pediatric resuscitation team along with elements of good team dynamics. Median (IQR) scores for individual items and total scores improved significantly following the intervention. The total Team OSCE score increased from a pre-intervention median of 6.25 (2) to a post-intervention median of 17 (2) (*p* < 0.02), indicating significant enhancement in skills. Comparison of pre and post intervention TPOT scores revealed a marked improvement in team performance through the iterative process of RCDP. There was a significant change in domain specific TPOT scores, with greatest gains in team structure and leadership.

Inter rater reliability of the two independent raters evaluating team performance using the Team OSCE and TPOT tools were assessed. Inter-rater reliability for the binary-rated Team OSCE items was assessed using Cohen’s Kappa. For the pre-intervention OSCE scores, the overall Kappa was 0.80, indicating substantial agreement. Post-intervention OSCE scores showed a similar level of agreement with a Kappa of 0.76. For the ordinal TPOT scores, inter-rater reliability was assessed using the Intraclass Correlation Coefficient (ICC). The pre-intervention ICC was 0.94 (95% CI: 0.60–0.98), and the post-intervention ICC was 0.93 (95% CI: 0.77–0.98), indicating good agreement throughout the study. Consensus scores were used for the final analysis to minimize rater-related bias. Pre- and Post- Intervention Team OSCE and TPOT scores have been summarized in Table 2.

**Table 2:** Comparison of Pre- and Post-Intervention team OSCE and TPOT scores

**Team OSCE**	**Pre-intervention Median (IQR)**		**Post-intervention Median (IQR)**	***p*** **-value** ^*^
Total Score		6 (2.25)	17 (2)	< 0.002
**Team Performance Observation Tool**				
**Domain Specific Scores**	**Pre-intervention Median (IQR)**		**Post-intervention Median (IQR)**	***p*** **-value** ^*^
Team structure		1 (0.08)	3.33 (0.33)	0.002
Communication		1 (0.56)	3 (0.56)	0.002
Leadership		1 (0.2)	3.1 (0.55)	0.002
Situational Monitoring		1 (0)	2.75 (0.56)	0.002
Mutual Support		1.75 (1)	3.5 (0.5)	0.002

*n* = 12

*Wilcoxon Signed Rank Test

### Self-perceived confidence

A marked improvement was observed in learners’ self-perceived confidence across both individual critical pediatric CPR related skills and interprofessional team collaboration. Significant improvement in confidence levels related to key resuscitation skills such as airway management, high-quality chest compressions, and effective bag-mask ventilation, was noted. Additionally, learners reported enhanced confidence in executing collaborative behaviors including role clarity, closed-loop communication, and shared decision-making during pediatric emergencies. This has been summarized in Table 3.

**Table 3:** Comparison of Pre- and Post-Intervention self-perceived confidence of participants using paired likert scale data

Confidence in	Pre-intervention Median (IQR)	Post-intervention Median (IQR)	*p*-value*
Initial assessment of an unresponsive child	2.5 (2.25)	4 (1)	< 0.001
Recognition of cardiac arrest in children	2 (1.25)	4 (1)	< 0.001
Airway assessment and basic airway management in children	3 (2)	4 (0)	< 0.001
Initiating high-quality chest compressions in pediatric patients	3 (2.25)	4 (1)	< 0.001
Recognizing cardiac arrest rhythm	3 (3)	4 (1)	< 0.001
Administering emergency medications in pediatric CPR	2 (3)	4 (1)	< 0.001
Understanding of Pediatric Cardiac Arrest Management Algorithm	2 (3)	4 (1)	< 0.001
Delegating and accepting tasks appropriately during emergencies	2 (3)	4 (1)	< 0.001
Communicating effectively with team members during pediatric CPR	3 (2)	4 (1)	< 0.001
Participating confidently in interprofessional resuscitation teams	2.5 (3)	4 (1)	< 0.001

*n* = 72

*Wilcoxon Signed Rank Test

### Feedback

The median agreement score (IQR) for the domain of session content was 4.8 (0.6), indicating a high level of perceived clarity and organization. Similarly, domains focusing on interprofessional collaboration development, skill acquisition, and overall educational value showed median scores > 4, reflecting strong learner endorsement. Summary of participant’s feedback is summarised in Table 4.

**Table 4:** Summary of participant feedback

Participant Feedback Scale: 1 = Strongly Disagree 2 = Disagree 3 = Neutral 4 = Agree 5 = Strongly Agree	Median (IQR)
Session Content	
Learning objectives were clearly defined	5 (0)
Session provided relevant knowledge in acute pediatric care	5 (0)
Simulation scenario was realistic	4 (1)
Facilitators provided clear explanations and guidance	5 (0)
Session promoted effective learning and skill development	5 (0)
Interprofessional Collaboration	
Session enhanced my understanding of the roles of different healthcare professionals	5 (1)
Session promoted effective communication among team members	5 (1)
Session emphasised teamwork in healthcare	5 (1)
Session promoted interprofessional collaboration	5 (1)
Debriefing helped me understand areas for improvement	5 (1)
Skill Development	
Session improved my clinical decision-making skills in acute pediatric care	5 (1)
Session increased my confidence in managing pediatric emergencies	4.5 (1)
Hands-on experience during simulation reinforced my knowledge	5 (1)
Session improved my ability to handle stress in acute scenarios	4 (1)
Simulation improved my problem-solving skills in acute pediatric care	5 (1)
Educational Value	
I would recommend this training to my colleagues	5 (0)
I found the session engaging	5 (0)
Adequate resources were provided for learning	4 (1)
Session length was appropriate	5 (1)
I would like to participate in similar interprofessional sessions	5 (0)

n = 72

## Discussion

This educational intervention represents a novel approach to interprofessional simulation education for undergraduate medical and nursing students, where RCDP methodology is utilized not just for skills training but also for practicing team performance within TeamSTEPPS framework. The findings suggest that this approach effectively improves knowledge, skills, team performance, and self-perceived confidence among novice learners.

The significant improvement in MCQ test and Team OSCE scores demonstrates the educational value of SBE. The observed gains in knowledge, clinical skills and teamwork competencies are consistent with findings from uniprofessional SBE research. Studies among nursing students have demonstrated that structured SBE improves knowledge and clinical judgement [6, 8, 20]. Our findings echo the results of a metanalysis by Cheng et al. evaluating simulation-based training in pediatric scenarios reported large pooled effect sizes for outcomes of knowledge and skills [21]. This alignment underscores the strength of SBE as a pedagogical modality for experiential learning. The present study extends the evidence from single profession cohorts, demonstrating comparable educational gains in interprofessional setting as well. The improvement in components of team dynamics is in consonance with past research recommendations that SBE in interprofessional settings provides more opportunities for honing interprofessional collaborative skills [22]. Our findings echo the significant improvements in teamwork and communication skills of medical and nursing students through IPSE documented in a recent systematic review and metanalysis [10]. This study adds to a growing body of evidence where interprofessional team training has been used for training in acute pediatrics focusing on resuscitation [23] or pediatric emergencies [24]. With their focus on teamwork and collaborative practice, IPSE programs have often incorporated the structured TeamSTEPPS framework for enhancing team performance [25]. Our findings are consistent with evidence favoring TeamSTEPPS-enhanced interprofessional simulation [15]. Studies utilizing TeamSTEPPS framework in IPSE for undergraduate learners also have demonstrated improved teamwork and communication skills [26, 27]. Similar to our study, albeit with more advanced uniprofessional learners, Goncalves et al. used TeamSTEPPS framework for team training in pediatric resuscitation with improvement in both technical and teamwork competencies [28] while Mayer et al. used this framework for training interprofessional teams working in pediatric intensive care units [29].

The educational design of IP-STAR intervention was grounded in principles of deliberate practice and mastery learning. McGaghie and colleagues described mastery learning as a form of competency-based education wherein learners achieve a stipulated standard of performance through deliberate practice [30]. Similarly, Ericsson postulated that expert performance emerges from individualized practice with immediate feedback and opportunities for successive refinement that transforms performance from competence to expertise [31]. These educational theories form the conceptual framework for RCDP which is employed in this study. This simulation based instructional strategy is designed to address high risk, low frequency clinical events and has been employed across various studies in resuscitation training [32]. RCDP allows novice learners who are still developing basic clinical skills, multiple opportunities at practicing segments of a complex scenario in a supportive learning environment. A high degree of synergism has been reported between principles of deliberate practice and Interprofessional Education Collaborative (IPEC) competencies [33]. Hence, multiple studies have evaluated role of RCDP in interprofessional settings with positive outcomes in terms of teamwork, crisis resource management, appropriate escalation of care and completion of time sensitive tasks [13, 34–36]. Our results parallel the findings of Colman et al. [13], who compared RCDP with traditional reflective debriefing across interprofessional emergency teams and found superior teamwork performance and retention of non-technical skills in the RCDP group. While Colman et al. focused on postgraduate practitioners, the present study demonstrates similar benefits at the undergraduate level, emphasizing that early exposure to RCDP-based IPE can cultivate foundational teamwork and leadership behaviors essential for later clinical practice.

There is consistent evidence that teamwork and leadership trainings improve team performances during CPR [37], and International Liaison Committee on Resuscitation (ILCOR) recommends team and leadership training should be included in advanced life support trainings [38]. A key strength of this project is therefore the novel integration of RCDP’s immediate feedback cycles with TeamSTEPPS’ framework. This integration provides a structured approach with clear guidance on effective team behaviors which the learners can internalize through RCDP with immediate correction and reinforcement. The improvement in TPOT domain specific scores for leadership and situational monitoring mirrors findings from a study in which RCDP improved adherence to neonatal resuscitation protocol and clinical decision-making skills [39].

Another important objective of this study was to address the challenge of educational silos of traditional curricula, by bringing nursing and medical students together in a collaborative learning environment. The simulation scenario and assessment tools required learners to work as a team with RCDP debriefs focused on technical skills as well as non-technical aspects of team performance such as role clarity, effective communication, mutual respect and collaboration. This approach aligns with IPE principle of learning “with, from and about each other” [4] and thus, fills a notable void in RCDP literature, which has predominantly focused on single-discipline cohorts for technical skills training. Further, the study postulates that exposure amongst novice undergraduate learners in their formative years may help prevent development of professional stereotypes impeding effective teamwork and collaborative practice upon graduation. The positive outcomes documented in this study suggest that IPE can be effectively implemented earlier in healthcare education than has traditionally been the case. These findings support early introduction of interprofessional collaboration rather than waiting until students have fully formed their professional identities. It is also important to note the favorable participant feedback received in this study which reflects the value students place on IPSE opportunities. This appreciation for interprofessional learning opportunities is consistent with findings from other studies of undergraduate IPE initiatives [26, 40]. The positive feedback for this IPSE module also emanates from the fact that it had features such as feedback, repetitive practice, physical realism of in-situ simulation which make simulation training effective [41].

The practice recommendations emanating from our findings is for curriculum designers and educators to consider embedding short, high-frequency RCDP-based interprofessional simulation sessions within undergraduate curricula to strengthen teamwork, communication, and crisis management competencies early in training. Faculty development in RCDP and TeamSTEPPS principles would further enhance implementation fidelity.

### Limitation

Despite its promising findings, we acknowledge several limitations of this study. The one-group pre-post design, without a control group, precludes the attribution of the observed improvements directly to the intervention. We also acknowledge that observed large positive effects in knowledge, skills, and team performance may be attributed to having novice learners as participants who benefited from the opportunity of deliberate practice based on immediate feedback from instructors because these novice learners start with a lower baseline and have more room for improvement compared to more experienced learners.

This study documented a marked increase in self-perceived confidence among learners in consonance with similar findings of confidence enhancement after SBE in uniprofessional settings and has been interpreted as reflection of both the opportunity of skill acquisition and psychological safety that the controlled environs of SBE provide [6]. However, this effect requires critical scrutiny in case of novice learners to account for metacognitive miscalibration explained in the Dunning – Kruger effect, whereby learners with limited baseline competence may overestimate their proficiency after a brief, positive learning experience [42]. However, it is also pertinent to point out that in this study, the observed increase in confidence of learners was accompanied by a statistically significant increase in both knowledge and demonstrable team performance. This triangulated improvement suggests that increase in confidence may be due to genuine skill acquisition rather than a perceptual overestimation.

It is equally important to highlight the fact that the post intervention assessment was conducted immediately after training session without a longer follow-up to assess the retention of knowledge and skills of the learners. Evidence suggests skills acquired through simulation training including teamwork competencies, decay without reinforcement [44]. Also, the transfer of skills and teamwork behaviors from the simulation environment to clinical practice was not assessed in this project. This lacuna will require redressal through future studies including follow-up assessments to evaluate durability of learning outcomes.

The biprofessional composition of the interprofessional teams – comprising only medical and nursing undergraduates - in this study constrains its ecological validity. Emergency response teams in real clinical environs would typically include a much wider spectrum of professionals such as emergency technicians, pharmacists, respiratory therapists, medical-social workers etc. whose roles and interactions would significantly affect the team dynamics. We acknowledge the possibility that the biprofessional setting may have simplified communication and role negotiation, potentially amplifying the observed positive effects on teamwork and performance. Future iterations should incorporate broader multicomponent teams to assess whether benefits noted in this study are maintained in authentic, ecologically diverse practice environment.

## Conclusion

This study provides evidence that integrating Rapid Cycle Deliberate Practice (RCDP) with the TeamSTEPPS framework in interprofessional simulation training effectively enhances undergraduate medical and nursing students’ knowledge, clinical skills, teamwork, and confidence in managing high-stakes pediatric resuscitation scenarios.

The integration of RCDP and TeamSTEPPS offers a scalable and resource-efficient model for interprofessional education, particularly suitable for low- and middle-income settings where high-technology simulation infrastructure may be limited. For validating this model’s scalability and durability, future research should incorporate more interprofessional teams, comparative designs, and longitudinal assessments to assess transfer of learnings to clinical practice.

## Supplementary Information

Supplementary Material 1.

Supplementary Material 2.

## Data Availability

Data is provided within the manuscript or supplementary information files.

## Abbreviations

AHRQ: Agency for Healthcare Research and Quality
BSc: Bachelor of Science
CIHC: Canadian Interprofessional Health Collaborative
CPR: cardiopulmonary resuscitation
ICC: Intraclass Correlation Coefficient
ILCOR: International Liaison Committee on Resuscitation
IPE: Interprofessional education
IPEC: Interprofessional Education Collaborative
IPSE: Interprofessional simulation education
IP: STAR-Interprofessional Simulation Training in Acute Pediatrics using Rapid Cycle Deliberate Practice
IQR: interquartile range
LMIC: Low-and Middle-Income Countries
MBBS: Bachelor of Medicine, Bachelor of Surgery
OSCE: Objectively Structured Clinical Examination
PALS: Pediatric Advanced Life Support
RCDP: Rapid Cycle Deliberate Practice
SBAR: Situation-Background-Assessment-Recommendation
SBE: Simulation based education
s-CVI: scale content validity index
TeamSTEPPS: Team Strategies and Tools to Enhance Performance and Patient Safety
TPOT: Team Performance Observation Tool
WHO: World Health Organization
