# Supplementary material for: Interprofessional simulation training in acute pediatrics using rapid cycle deliberate practice (ip-star)
Source: BMC Med Educ. 2025 Dec 29;25:1729. doi: 10.1186/s12909-025-08302-4 (PMC12750579; doi:10.1186/s12909-025-08302-4)
Supplement: Supplementary file 1 — Supplementary Material 1. [file 12909_2025_8302_MOESM1_ESM.docx]

**Additional File 1**

**IP-STAR Simulation Scenario Script**

| **Simulation Scenario Title:** Pediatric Cardiac Arrest, Pulseless Electrical Activity, Hypovolemic Shock  **Simulation Training Methodology:** Rapid Cycle Deliberate Practice  **Learners:** MBBS & BSc Nursing Students | | | |
| --- | --- | --- | --- |
| **Pre-brief** (for all rounds): Lay down “basic assumptions”, ensure psychological safety, establish confidentiality contract, promote learners to suspend disbelief. Introduce participants to manikin, equipment, monitor (with normal parameters) and set-up. | | | |
| **Manikin:** Sim baby or similar manikin dressed as an 8-month-old infant  **Clinical setting:** Pediatrics Emergency Room  **Diagnosis**: Cardiac Arrest (pulseless electrical activity) | | | |
| **Equipment**: | Airway and Breathing: Guedel airway, Yankeur suction set, portable suction, suction catheter size 6, 8. NG tube 8 size Infant oxygen mask with tubing, Self inflating bag with reservoir and mask, oxygen supply, tubing for oxygen, Neonatal Laryngoscope, ET tubes 3.5, 4, 4.5, tapes to fix ET, saturation probe,  Circulation: ECG leads, Stethoscope and BP cuff. IV fluids NS, DNS, 0.45% DNS, 10% Dextrose. IV stand, Syringes 1 2,5, 10, 20 50 ml, Cannula yellow, 3-way stopcock with extension set. Tapes to fix. syringe pump 1 with IV extension set. BM/IO line. (ALL can be used ones-gas sterilized). Defibrillator.  c) Drugs: Adrenaline, NS flush, (can be empty vials/ syringes with label, filled with water) *“All medications & IV fluids should be marked “FOR SIMULATION PURPOSE ONLY: NOT FOR HOSPITAL USE”*  d) Other: clothes for manikin. Crash cart, sanitizer, gloves, Ice pack, drainage setup for IV on manikin | | |
| **Simulation Scenario** | 8-month-old Ravi has been brought to Pediatric ER by his mother with complaints of diarrhea and vomiting for 2 days. Mother reports that Ravi has refused feed since this morning and is having rapid breathing. Since morning today she has noted that Ravi is becoming progressively difficult to wake and has become unresponsive while coming to ER in ambulance. | | |
| **Additional information for facilitator** | Sign and symptoms: unresponsive, no breathing, no pulse  No allergies.  Paracetamol has been administered 4 hours back  No significant past h/x. Uneventful perinatal and developmental h/x  Last feeds 8 hours back  Weighs 8 kg  If team asks for more history at any time, mother is inconsolable and cannot talk.  If team asks for glucose, provide report of glucose = 44 mg/dL, 60 seconds after request is made.  If team asks for blood gas, inform machine is not working in ER  If team asks for temperature, provide temp =98.4^o^F 10 seconds after request is made.  For all other inquiries, reply that the results will be delayed.  (Vital signs not read aloud, assessed on mannequin and given on monitor once attached)  **Guidance for RCDP for expected actions**  **Hard stops:** Actions which must be performed in the recommended way before scenario progresses specially linked to primary learning objectives of each round. If the teams failed to demonstrate following - asepsis, call for assembling a team, role allocation, clear messages, monitoring of patient status and mutual respect; it will trigger a hard stop.  **Soft stops:** Actions where variability of performance is allowed related to team dynamics, communication and sharing of mental model  Use RCDP for Hard Stops and address Soft Stops through RCDP if time permits or address them through final debrief at the end of round  Facilitators will also use [TeamSTEPPS Team Performance Observation Tool](https://www.ahrq.gov/sites/default/files/wysiwyg/teamstepps-program/tools/ts-team-performance-tool.pdf) to monitor team performance and will debrief at the end of each round | | |
| **Embedded participants** | Mother (Round 1); Senior Resident (Round 5) | | |
| **Embedded Participant Script** | Mother – *“Ravi has been having fever and cough for past 3 days. We gave him Paracetamol and some cough syrup. However, since yesterday, he has been refusing feeds and is having rapid breathing. Since today morning she has been very lethargic and is difficult to wake up. Now he has stopped crying while coming to the hospital”*  Mother will continue to be anxious and desperate till she is empathetically addressed by the doctor and asked to wait in the room outside.  Senior Resident: *“Please brief me about this case”* | | |
| **Primary Learning Objectives:** | **Round 1**   1. Call for help 2. Recognition of cardiac arrest 3. Immediate initiation of CPR 4. Role allocation 5. Attachment of monitor/defibrillator   **Round 2:**   1. High quality CPR 2. Appropriate resuscitation ergonomics 3. Appropriate chest compressions 4. Adequate bag and mask ventilation   **Round 3:**   1. Administration of Epinephrine at the earliest and then every 3-5 mins 2. Identification of cardiac arrest rhythm 3. Follow PALS algorithm   **Round 4:**   1. Consider advanced airway placement 2. Look for modifiable causes – Hs & Ts; Correction of hypovolemia and hypoglycemia   **Round 5:**   1. After ROSC; oxygen through nonrebreather mask 2. Prepare to shift to PICU with SBAR handover   **All rounds:**   1. Use closed-loop communication 2. Clear messages addressed to specific individuals 3. Team members confirm each request is understood and close the loop 4. Demonstrate mutual respect with language and behavior 5. Use proper names with eye contact 6. Share ideas and information as constructive interventions 7. Provide a shared mental model (summary of scenario and next steps given to maintain situational awareness) 8. Adjust team roles to needs of scenario (leader and individuals) 9. Provide rapid, coordinated action 10. Actions recommended in [TeamSTEPPS Team Performance Observation Tool](https://www.ahrq.gov/sites/default/files/wysiwyg/teamstepps-program/tools/ts-team-performance-tool.pdf) | | |
| **Round 1: Assessment, Call for help, Role Allocation** | | | |
| Patient condition  (instructor will need to provide this information when learners simulate the action as manikin will not be able to show these) | | Simulator parameters | Facilitators notes |
| A: patent, minimal nasal secretions  B: Not breathing  C: No pulse  D: Unresponsive  E: cold periphery | | Show only after monitor is attached  HR: 00, RR:00, Rhythm: organised electrical activity BP: Not recordable Oxygen Saturation (O2Sat): 00 | 10 Seconds after calling, the remainder of team is allowed to enter room  If mother is not empathetically addressed, then she stays in scenario distraught and anxious |
| **Round 2: High Quality CPR** | | | |
| Patient condition  (instructor will need to provide this information when learners simulate the action as manikin will not be able to show these) | | Simulator parameters | Facilitators notes |
| A: patent  B: Not breathing  C: No pulse  D: Unresponsive  E: cold periphery | | HR: varying with chest compression, RR:00, Rhythm: organised electrical activity at rhythm checks BP: Not recordable Oxygen Saturation (O2Sat): 00 | Look for use of step stool and cardiac board |
| **Round 3: Epinephrine; Rhythm Check** | | | |
| Patient condition  (instructor will need to provide this information when learners simulate the action as manikin will not be able to show these) | | Simulator parameters | Facilitators notes |
| A: patent, minimal nasal secretions  B: Not breathing  C: No pulse  D: Unresponsive  E: cold periphery | | HR: varying with chest compression, RR:00, Rhythm: organised electrical activity at rhythm check BP: Not recordable Oxygen Saturation (O2Sat): 00 | Look for change of compressors during rhythm check |
| **Round 4: Advanced airway; Hs & Ts** | | | |
| Patient condition  (instructor will need to provide this information when learners simulate the action as manikin will not be able to show these) | | Simulator parameters | Facilitators notes |
| A: patent, minimal nasal secretions  B: Not breathing  C: No pulse  D: Unresponsive  E: cold periphery | | HR: varying with chest compression, RR:00, Rhythm: organised electrical activity at rhythm checks BP: Not recordable Oxygen Saturation (O2Sat): 00  After correction of hypoglycaemia and hypovolemia, convert to ROSC (Round 5) | Team should prepare to intubate or ask for help for intubation Team should consider Hs & Ts; identifying hypovolemia and hypoglycemia and correct it |
| **Round 5: Post resuscitation care** | | | |
| Patient condition  (instructor will need to provide this information when learners simulate the action as manikin will not be able to show these) | | Simulator parameters | Facilitators notes |
| A: patent  B: Regular breaths  C: Central and peripheral pulses palpable  D: Responds to pain  E: cold periphery | | HR: 160/min RR:30, Rhythm: sinus BP: 70/46 Oxygen Saturation (O2Sat): 90% | Introduce embedded participant as Senior Resident. Look for SBAR handover and plan to shift to ICU |

| *Scenario scripted by:*  *Dr Manish Kumar*  *Additional Professor, Dept of Paediatrics, AIIMS Gorakhpur & Fellow, MAHE – FAIMER International Institute for Leadership in Interprofessional Education, Manipal* |
| --- |
