# Supplementary material for: Interprofessional simulation training in acute pediatrics using rapid cycle deliberate practice (ip-star)
Source: BMC Med Educ. 2025 Dec 29;25:1729. doi: 10.1186/s12909-025-08302-4 (PMC12750579; doi:10.1186/s12909-025-08302-4)
Supplement: Supplementary file 2 — Supplementary Material 2. [file 12909_2025_8302_MOESM2_ESM.docx]

**Additional File 2**

**IP-STAR Pre/Post Test**

1. **According to PALS, what is the first step in assessing an unresponsive patient?**
   1. Start chest compressions
   2. Check pulse and breathing
   3. Give oxygen immediately
   4. Administer epinephrine
2. **What is the recommended depth of chest compressions for an infant according to PALS?**
   1. At least one-third of the anteroposterior chest diameter
   2. At least one-fourth of the anteroposterior chest diameter
   3. At least one-half of the anteroposterior chest diameter
   4. 5 cm regardless of age
3. **A child is in pulseless ventricular tachycardia (pVT). After initiating CPR and giving the first shock, what is the next medication recommended by PALS?**
   1. Amiodarone 5 mg/kg IV/IO
   2. Epinephrine 0.01 mg/kg IV/IO
   3. Atropine 0.02 mg/kg IV/IO
   4. Sodium bicarbonate 1 mEq/kg IV/IO
4. **What is the recommended initial dose of epinephrine during pediatric cardiac arrest in PALS?**
   1. 0.01 mg/kg IV/IO (1:10,000 concentration)
   2. 0.1 mg/kg IV/IO (1:1,000 concentration)
   3. 1 mg IV/IO regardless of weight
   4. 0.1 mg IV push
5. **According to PALS, what is the recommended initial energy dose (in J/kg) for defibrillation in paediatric patients with shockable rhythms (VF/pVT)?**
   1. 1
   2. 2
   3. 4
   4. 10
6. **Which of the following is the most common cause of pediatric cardiac arrest according to PALS?**
   1. Primary cardiac arrhythmia
   2. Respiratory failure leading to hypoxia
   3. Sudden cardiac death from congenital defects
   4. Myocardial infarction
7. **During paediatric advanced airway management, what is the recommended respiratory rate for a child receiving rescue breaths with a pulse?**
   1. 10-12 breaths per minute
   2. 20-30 breaths per minute
   3. 12-20 breaths per minute
   4. 6-8 breaths per minute
8. **Identify this cardiac arrest rhythm?**

**
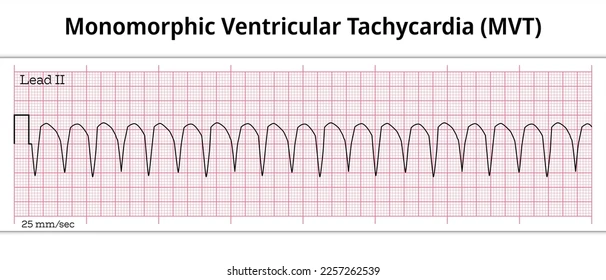
**

- 1. Supraventricular Tachycardia
  2. Ventricular Tachycardia
  3. Ventricular Fibrillation
  4. Atrial Fibrillation

1. **Which of the following is a shockable rhythm?**
   1. Pulseless Ventricular Tachycardia
   2. Ventricular Fibrillation
   3. Both
   4. None
2. **What does SBAR stand for in the context of healthcare communication?**
   1. Safety, Background, Assessment, Response
   2. Status, Briefing, Assessment, Report
   3. Summary, Background, Analysis, Review
   4. Situation, Background, Assessment, Recommendation

**IP-STAR Team OSCE Evaluation Tool:**

|  | **Action** *(1 Point per Item)* | **☑ / ⮽** |
| --- | --- | --- |
| 1 | Call for help |  |
| 2 | Recognition of cardiac arrest |  |
| 3 | Immediate initiation of CPR |  |
| 4 | Role allocation |  |
| 5 | Attachment of monitor/defibrillator |  |
| 6 | Appropriate resuscitation ergonomics |  |
| 7 | Appropriate chest compressions |  |
| 8 | Adequate bag and mask ventilation |  |
| 9 | Administration of Epinephrine at the earliest and then every 3-5 mins |  |
| 10 | Identification of cardiac arrest rhythm |  |
| 11 | Follow PALS algorithm |  |
| 12 | Consider advanced airway placement |  |
| 13 | Look for modifiable causes – Hs & Ts |  |
| 14 | After ROSC; oxygen through nonrebreather mask |  |
| 15 | Gives SBAR handover |  |
| 16 | All messages or orders addressed to specific individuals |  |
| 17 | Gives Clear Messages |  |
| 18 | Uses closed-loop communication |  |
| 19 | Shows Mutual respect |  |
| 20 | Intervenes in a constructive manner |  |

**IP-STAR Self-perceived Learner Confidence**

**Instructions**:
Please rate your **confidence** in the following aspects of acute pediatric care using the 5-point Likert scale below.

**Scale**:
1 – Not confident at all
2 – Slightly confident
3 – Moderately confident
4 – Very confident
5 – Extremely confident

| **Competency** | **Confidence rating** |
| --- | --- |
| Initial assessment of an unresponsive child |  |
| Recognition of cardiac arrest in children |  |
| Airway assessment and basic airway management in children |  |
| Initiating high-quality chest compressions in pediatric patients |  |
| Recognizing cardiac arrest rhythm |  |
| Administering emergency medications in pediatric CPR |  |
| Understanding of Pediatric Cardiac Arrest Management Algorithm |  |
| Delegating and accepting tasks appropriately during emergencies |  |
| Communicating effectively with team members during pediatric CPR |  |
| Participating confidently in interprofessional resuscitation teams |  |

**IP-STAR Participant Feedback Survey**

**Instructions:** Please indicate your level of agreement with the following statements by selecting the most appropriate response.

**Scale:** 1 = Strongly Disagree 2 = Disagree 3 = Neutral 4 = Agree 5 = Strongly Agree

1. **Session Content & Delivery**

| Learning objectives were clearly defined.  Session provided relevant knowledge in acute pediatric care  Simulation scenario was realistic  Facilitators provided clear explanations and guidance  Session promoted effective learning and skill development. |  |
| --- | --- |

1. **Interprofessional Collaboration & Teamwork**

| Session enhanced my understanding of the roles of other healthcare professionals  Session promoted effective communication among team members.  Session emphasised teamwork in healthcare  Session promoted interprofessional collaboration  Debriefing helped me understand areas for improvement. |  |
| --- | --- |

1. **Skill Development**

| Session improved my clinical decision-making skills in acute pediatric care  Session increased my confidence in managing pediatric emergencies  Hands-on experience during simulation reinforced my knowledge.  Session improved my ability to handle stress in acute scenarios  Simulation improved my problem-solving skills in acute pediatric care. |  |
| --- | --- |

1. **Educational Value**

| I would recommend this training to my colleagues.  I found the session engaging  Adequate resources were provided for learning.  Session length was appropriate  I would like to participate in similar interprofessional sessions |  |
| --- | --- |
